# Association of Diabetes in Pregnancy with Child Weight at Birth, Age 12 Months and 5 Years – A Population-Based Electronic Cohort Study

**DOI:** 10.1371/journal.pone.0079803

**Published:** 2013-11-13

**Authors:** Kelly Morgan, Mohammed Rahman, Mark Atkinson, Shang-Ming Zhou, Rebecca Hill, Ashrafunnesa Khanom, Shantini Paranjothy, Sinead Brophy

**Affiliations:** 1 College of Medicine, Swansea University, Swansea, Wales, United Kingdom; 2 School of Medicine, Cardiff University, Cardiff, Wales, United Kingdom; University of Missouri-Kansas City, United States of America

## Abstract

**Background:**

This study examines the effect of diabetes in pregnancy on offspring weight at birth and ages 1 and 5 years.

**Methods:**

A population-based electronic cohort study using routinely collected linked healthcare data. Electronic medical records provided maternal diabetes status and offspring weight at birth and ages 1 and 5 years (n = 147,773 mother child pairs). Logistic regression models were used to obtain odds ratios to describe the association between maternal diabetes status and offspring size, adjusted for maternal pre-pregnancy weight, age and smoking status.

**Findings:**

We identified 1,250 (0.9%) pregnancies with existing diabetes (27.8% with type 1 diabetes), 1,358 with gestational diabetes (0.9%) and 635 (0.4%) who developed diabetes post-pregnancy. Children whose mothers had existing diabetes were less likely to be large at 12 months (OR: 0.7 (95%CI: 0.6, 0.8)) than those without diabetes. Maternal diabetes was associated with high weight at age 5 years in children whose mothers had a high pre-pregnancy weight tertile (gestational diabetes, (OR:2.1 (95%CI:1.25–3.6)), existing diabetes (OR:1.3 (95%CI:1.0 to 1.6)).

**Conclusion:**

The prevention of childhood obesity should focus on mothers with diabetes with a high maternal pre-pregnancy weight. We found little evidence that diabetes in pregnancy leads to long term obesity ‘programming’.

## Introduction

Diabetes in pregnancy is linked to complications in 2–5% of all pregnancies [1] and is commonly recognised as a modifier of growth and development of the foetus, with suggestions of long term ‘programming’ through fuel-mediated teratogenesis [2]. Obesity currently affects 40 million children under the age of 5 years worldwide [3]. As the number of infants exposed to diabetes in pregnancy increases [4], it is important to establish whether an adverse hyperglycaemic intrauterine environment represents a long term risk factor for overweight. Immediate postnatal differences have been demonstrated amongst offspring of mothers with diabetes, with reports of macrosomia [5], increased adiposity and neonatal hypoglycaemia [6]. Similar birth outcomes have also been shown amongst offspring exposed to high maternal glucose levels below the diabetes threshold [7].

Investigations into whether these adverse effects persist into later infancy and childhood are scarce and show inconsistent findings. Previous studies focusing on the Pima Indian population have produced strong findings of an independent effect of exposure to maternal diabetes on offspring obesity [8], whilst studies amongst other populations have produced mixed findings [9], [10]. Furthermore despite previously reporting adverse birth outcomes, a study [11] based on 15-centres worldwide did not find an association between varying levels of maternal glycaemia (below the diabetic threshold) and obesity at age 2 years. Two recent systematic reviews [12], [13] also highlight the inconsistency of research whilst demonstrating the importance of controlling for maternal pre-pregnancy BMI. In both reviews, the association between maternal diabetes and childhood obesity was attenuated when maternal BMI was accounted for in the analysis. These findings raise the question as to whether future preventative strategies for childhood obesity should focus on maternal pre-pregnancy weight.

We carried out a large-scale record linked database analysis of health data to investigate the association between existing diabetes, gestational diabetes and maternal diabetes developed post pregnancy (a marker for exposure to poor lifestyle factors rather than diabetes) and offspring being large (above the 90^th^ centile) at birth, age 12 and age 60 months. A second aim was to examine whether any observed association was independent of maternal pre-pregnancy BMI, age and smoking status.

## Materials and Methods

### Study design and participants

The study population consisted of all women with GP (General Practitioner) data (registered longer than 12 month period) and a corresponding pregnancy available in the National Community Child Health Database (NCCHD). Due to the nature of our study design ethical approval and participant consent was waived by the approving IRB (Institutional Review Board), the Information Governance Review Panel (IGRP). Mother-child pairs were identified using delivery records on the NCCHD, a resource encompassing child health data from Local Health boards throughout Wales. Mothers of twins were included in the study as two separate mother-child pairs (Mum –Twin 1 and Mum- Twin 2). Mothers were also duplicated if more than one child was born throughout the designated time frame. To ensure we had a complete record of the pregnancy and birth period, only records where the mother was registered with a GP for the 12 month period before the birth were selected. The use of electronic data to identify diabetes diagnosis has been examined by others [14]. We used both read codes, medications prescribed and glucose test results as outlined in a recent publication [14] to ensure a reliable diagnosis. General Practitioner and Brecon Group records (Welsh register of individuals who have been diagnosed with diabetes throughout childhood) were searched for maternal diabetes diagnosis codes to categorise mothers into one of the following groups: *no diabetes, existing diabetes, gestational diabetes*, and *diabetes diagnosed post pregnancy*. Offspring data included weight measures at birth and ages 1 and 5 years. A more detailed account of explanatory and outcome variables is provided in Table S1.

### Setting

Offspring weight and maternal diabetes status were examined using linkages within the Secure Anonymised Information Linkage (SAIL) databank [15], [16]. The SAIL databank anonymously record-links routinely collected data held in healthcare and social datasets at the Health Information Research Unit (HIRU), Swansea University, UK. For each dataset within the SAIL databank, an individual is assigned an Anonymised Linking Field (ALF_E), based on their names, addresses or National Health Service (NHS) numbers, which is used to link across datasets. Currently, the WECC (Wales Electronic Cohort for Children) dataset consists of 804,290 children born over a 19-year time period (1990 to 2008). All data within the SAIL gateway is treated in accordance with the Data Protection Act 1998. To date the SAIL databank incorporates over 2.5 billion records from multiple health and social care events and currently involves 41% (198/484) of the GP Practices across Wales providing data to SAIL. As a result 908,805 (29%) out of 3,147,416 individuals registered with a GP practice in Wales have accessible electronic records of which 49.9% are females with a mean age of 39.8 years (in both the total population and the SAIL sample).

### Statistical analyses

All statistical analyses were performed using STATA version 12 (STATA, Texas, USA). Differences in distributions of baseline characteristics (maternal and offspring) in relation to maternal diabetes status were analysed using *t* tests for continuous variables and χ^2^ for categorical variables. We used binary logistic regression to calculate odds ratios for offspring being large- (weight above the 90^th^ percentile for gestational age) or small for gestational age (weight below the 10^th^ percentile for gestational age) at birth and large at ages 12 and 60 months according to maternal diabetes status. Large at ages 12 and 60 months was classified using age- and gender-specific percentiles based on the UK 1990 Growth charts [17] with reference to weight data. Thirdly, we used logistic regression models to examine associations between maternal diabetes status with offspring size adjusted for maternal pre-pregnancy weight, age and smoking status. Associations did not differ by infant gender therefore all results are reported with males and females combined.

After reporting on complete-case data we conducted sensitivity analysis by imputing missing data for mother weight and weight of the child at age 5 years through a process of multiple imputations. Mother-child pairs with missing data were more likely to be deprived whilst some missing at random depend on data captures of weight at school entry and school rather than any difference between child weights. In our analysis, the multiple sets of point estimates for previous weights and their standard errors were combined to obtain a single point estimate. The MICE (Multivariate imputation by chained equations [1]) package in Stata 12 was used to implement this analysis procedure, in which the predictive mean matching (pmm) imputation method together with a logarithm transformation was used to preserve the normality of the variables.

## Results

### Participants

**Figure 1:**
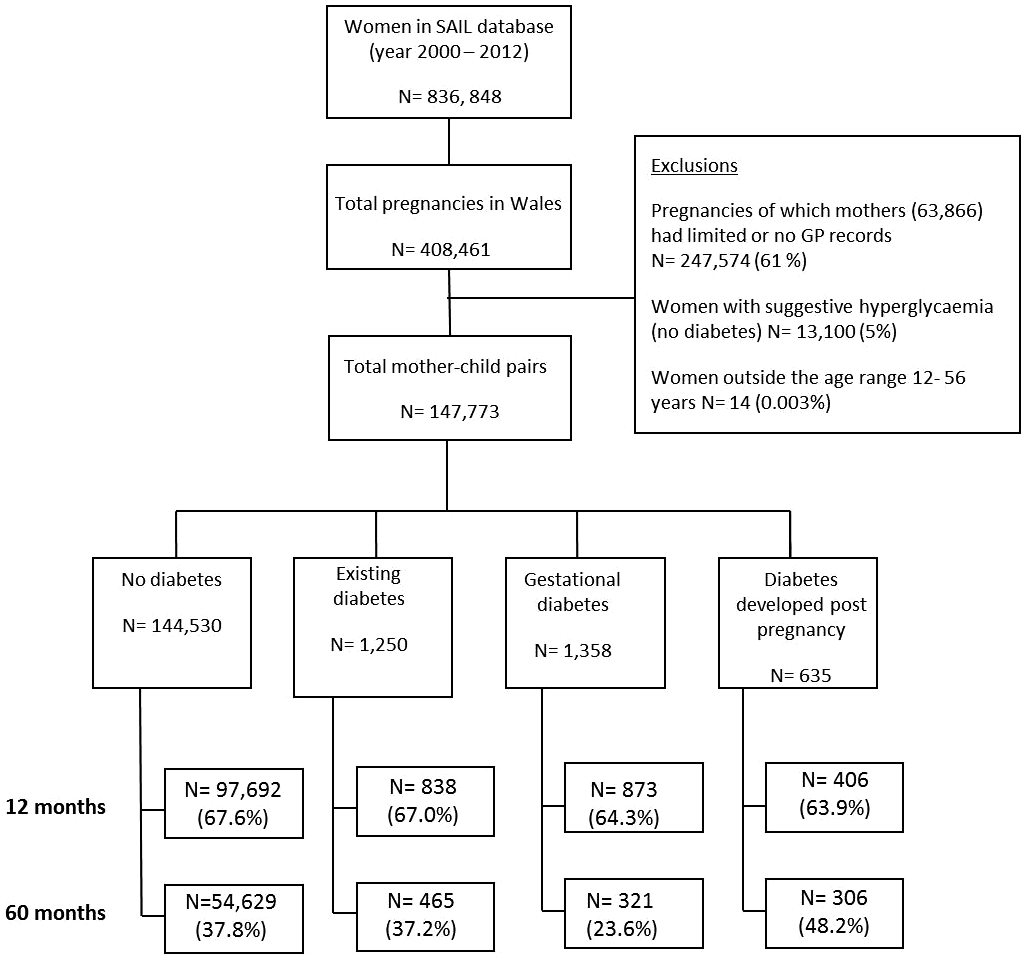
Participation flow diagram throughout the study and the formation of mother-pair groups.

There were 408,461 individuals born in Wales between the years 2000 and 2012 of which 160,887 (39.2%) had mothers with GP records for longer than a 12 month period around the child birth date. Those with high glucose values suggestive of hyperglycaemia that did not develop diabetes post pregnancy were removed and the remaining 147,773 women were analysed in this prospective longitudinal study (see Figure 1). Amongst the study population, there were 2,275 (1.5%) twin pregnancies. The mean birth weight of this sub group was 2.43 kg (SD 0.58) with an average gestational length of 35.6 weeks. Ninety seven percent of twin pregnancies occurred in the no diabetes group. Each mother-child pair was stratified by mothers’ diabetes status; 144,530 (97.8) were exempt from diabetes, 2,608 (1.8%) had diabetes during the course of the pregnancy (of which 52.1% had gestational diabetes) and 635 (0.4%) developed diabetes post pregnancy (12.2% of these women were hyperglycaemic during pregnancy). Of the women with pre-existing diabetes 27.8% had type 1 diabetes with a mean age of 11.3 years at diabetes onset. Women who had developed type 2 diabetes before pregnancy were diagnosed on average at 25.3 years and had a mean time between diabetes diagnosis and onset of pregnancy of 6.1 years. Of the women developing diabetes post pregnancy in our study the average time period from birth delivery to diabetes onset was 5 years (range 2.6 months - 11.6 years).

### Descriptive data

The average age of mothers at time of delivery was 28.3 years (range 12.6 to 51 years, see Table 1). Maternal pre-pregnancy BMI data was available for 58% (n =  85,056) of the sample and showed 43% of women as overweight (25.4%) or obese (17.6%). Mean pre-pregnancy weight values significantly differed across the four groups (p = 0.001) with the highest values displayed amongst mothers who developed diabetes post pregnancy (69.7 kg v 86.6 kg, *p*<0.0001). Smoking data revealed over a quarter of women (25.8%) smoked throughout pregnancy.

**Table 1:** Characteristics of the mother and offspring.

		No diabetes	Existing diabetes	Gestational diabetes	Diabetes developed post pregnancy
N	147,773	144,530	1,250	1,358	635
**Maternal data**					
Age at delivery (years)	147,773	28.3±6.1	30.5±6.1	31.7±5.8	29.5±6.2
WIMD score*	139,714				
1 (most affluent)		18.4(25,070)	16.0(190)	19.5(251)	13.1(80)
2		18.8(25,760)	16.7(199)	19.2(247)	13.6(83)
3		19.5(26,622)	20.9(247)	20.3(261)	20(122)
4		21.5(29,439)	21.1(251)	20.5(263)	24.9(152)
5 (most deprived)		21.8(29,741)	25.3(300)	20.5(263)	28.4(173)
Mulitparity (≥2 partus)	147,773	76.3(110,241)	71.8(898)	71.2(967)	81.6(518)
Smoking during pregnancy	73,864	25.7(19,014)	35.3(230)	30.3(178)	22.2(82)
Pregestational weight (kg)	91,101	68.2±16.2	79.8±21.8	82.2±22.5	86.6±22.1
Pre-pregnancy BMI>24.9 (kg/m2)	85,056	41.6 (35,125)	65.3 (589)	71.9 (655)	81.8 (240)
**Birth data**					
Male sex	147,773	52(64,463)	51 (633)	52(709)	51(325)
Birth weight (kg)	147,773	3.4±0.6	3.4±0.8	3.5±0.6	3.5±0.7
Gestational age (wks)	142,596	39.3±2.2	37.0±2.9	38.4±1.9	38.4±2.7
Preterm delivery (<37 weeks)	142,596	7.4 (10,412)	31.2(380)	10.7(143)	13.9(84)

Results are expressed as mean±SD or %(number) *Welsh Index of Multiple Deprivation (overall deprivation rank in Wales).

### Outcome data

Children were more likely to be born large if their mother had diabetes (mothers with existing diabetes (adjusted OR:2.96 (95%CI 32.7 to 38.3)), gestational diabetes (adjusted OR:1.82 (95%CI 25.1 to 30.0)) or if their mother developed diabetes post pregnancy (adjusted OR:1.42 (95%CI 23.6 to 30.1))) (See Table 2). These findings did not alter after adjusting for pre-pregnancy weight, smoking status or maternal age (See Table 3). At 12 months, children of mothers with existing diabetes were not at risk of being large in comparison to children born to mothers without diabetes in pregnancy. In fact, at age 1 year a lower proportion of children were categorised as large if mothers had existing diabetes (adjusted OR:0.62(95%CI 0.57 to 0.83)) when compared to those children born to mothers with no diabetes. Further stratifying mothers into three pre-pregnancy weight categories (lower, middle and upper tertiles) revealed that women with type 1 diabetes who were in the lowest weight tertile (OR: 0.6 (95% CI:0.4 to 1.0)) or the upper weight tertile (OR: 0.6 (95%CI:0.4 to 0.8)) were less likely to have a large child at 12 months compared to those without diabetes. Unadjusted analysis suggested children born to mothers with gestational diabetes were larger at 5 years (adjusted OR:1.9 (95%CI 1.4 to 2.4)). However, once this comparison was adjusted for pre-pregnancy weight, age and smoking status, the association was attenuated. Further examining the relationship between maternal weight and gestational diabetes showed that children with mothers who had gestational diabetes and were in the middle weight tertile (OR: 2.14 (95%CI:1.2 to 3.8)) or highest weight tertile (OR: 2.1 (95%CI:1.25 to 3.6)) were more likely to be large at age 5 years (see Table 4).

**Table 2:** Prevalence of large offspring at birth, age 1 and age 5 years, stratified by mother’s diabetes status during or after pregnancy.

	No diabetes (95%CI)	Existing diabetes (95%CI)	Gestational diabetes (95%CI)	Diabetes developed post pregnancy (95%CI)
N	144,530	1,250	1,358	635
Small for gestational age	2.13% (3,004)	1.88% (21)	1.07% (14)	2.18% (13)
	(2.1 to 2.2)	(1.2 to 2.8)	(0.6 to 1.8)*	(1.28 to 3.69)
Large for gestational age	12.6% (17,718/140,184)	35.5% (396/1117)	27.4% (360/1312)	27.0% (161/596)
	(12.5 to 12.8)	(32.7 to 38.3)*	(25.1 to 30.0)*	(23.6 to 30.1)*
N	97,692	838	873	406
>90^th^ centile by age 12 months	20.6% (20,121)	15.2% (127)	20.6% (180)	20.0% (81)
	(20.4 to 20.9)	(12.9 to 17.8)*	(18.1 to 23.4)	(16.4 to 24.1)
N	54,269	465	321	306
>90^th^ centile at age 60 months	14.9% (8079)	17.6% (82)	24.6% (79)	16.3% (50)
	(14.6 to 15.2)	(14.4 to 21.4)	(20.2 to 29.6)*	(12.6 to 20.9)
N	81,302	753	561	482
>90^th^ centile at age 60 months including imputed data	17.2% (13,997)	23.2% (175)	25.5% (143)	25.9% (125)
	(17.0 to 17.5)	(20.4 to 26.4)*	(22.1 to 29.3)*	(22.2 to 30.0)*

**Table 3:** Regression analysis. Adjusted odds ratio for large size at birth, 12 and 60 months.

Risk factor	Large for gestation		Large at age 12 months		Large at age 60 months	
	Odd ratio	95%CI	Odd ratio	95%CI	Odd ratio	95%CI
Pre-pregnancy weight (per kg increase in weight)	1.02	1.0–1.0	1.01	1.0–1.0	1.02	1.0–1.0
Pre-existing diabetes	2.96	**2.4**–**3.6**	0.62	**0.5**–**0.8**	1.03	0.7–1.5
Gestational diabetes	1.82	**1.5**–**2.3**	0.93	0.7–1.2	1.10	0.6–1.9
Diabetes after pregnancy	1.42	**1.0**–**2.1**	0.49	**0.3**–**0.8**	0.57	0.3–1.1
Mothers age at delivery	1.02	1.0–1.0	0.99	1.0–1.0	1.00	1.0–1.0
Smoking in pregnancy	0.79	**0.7**– **0.8**	0.94	**0.9**–**1.0**	0.95	**0.9**–**1.0**

**Table 4:** Odds ratio for having a large infant at age 5 years stratified by prepregnancy weight tertile (comparisons of women with gestational diabetes mellitus versus non diabetes mothers).

Prepregnancy weight		Large child % (n)	Non large child % (n)	OR (95%CI)
Lower tertile	Gestational	15.3 (9)	84.7 (50)	
	No diabetes	8.7 (831)	91.3 (8,712)	1.89 (0.9–3.8)
Middle tertile	Gestational	27.1 (16)	72.9 (43)	
	No diabetes	14.8 (1,457)	85.2 (8,378)	2.14 (1.2–3.8)*
Higher tertile	Gestational	37.3 (22)	62.7 (37)	
	No diabetes	21.8 (2,022)	78.2 (7,235)	2.1 (1.25–3.6)*

### Sensitivity analysis

Imputation of missing values of weight at age 5 years based on previous child weight measures and maternal characteristics. Imputing missing weight values at age 5 years provided an extra 49% (n  = 27,385) of mother-child pairs for analyses. Binary logistic regression revealed that children born to mothers with any form of diabetes across the life span were at a significantly increased risk of being large at age 5 years. However, analysis adjusting for confounding factors (maternal age, smoking and pre-pregnancy weight) showed no effect of diabetes on infant weight at age 5 years (Table 5). Stratifying findings by maternal pre-pregnancy weight (Table 6) showed women in the highest weight tertile with existing (OR:1.3 (95%CI 1.0 to 1.6)) or gestational diabetes (OR:1.6 (95%CI 1.2 TO 2.1)) were more likely to have a large child at age 5 years when compared to the larger women without diabetes.

**Table 5:** Regression analysis.

Risk factor	Large at age 60 months	
	Odd ratio	95%CI
Pre-pregnancy weight (per kg increase in weight)	1.03	1.0–1.0
Pre-existing diabetes	1.16	0.9–1.5
Gestational diabetes	0.87	0.6–1.3
Diabetes after pregnancy	0.86	0.6– 1.3
Mothers age at delivery	0.99	1.0–1.0
Smoking in pregnancy	0.94	**0.9**– **1.0**

Adjusted odds ratio for large size at age 60 months using imputed values for age 60 months.

**Table 6:** Odds ratio for having a large infant at age 5 years stratified by prepregnancy weight tertile (original and imputed data).

Prepregnancy weight		Large child	Non large child	OR (95%CI)
		% (n)	% (n)	
Lower tertile	No diabetes	9.2 (1,472)	90.8 7(14,447)	1.00 (Ref)
	Existing	8.4 (7)	91.6 (76)	0.9 (0.4 to 2.0)
	Gestational	8.3 (5)	91.7 (55)	0.9 (0.4 to 2.2)
	Post pregnancy	12.5 (3)	87.5 (21)	1.4 (0.4 to 4.7)
Middle tertile	No diabetes	19.1 (2,619)	80.9 (13,682)	1.00 (Ref)
	Existing	16.9 (27)	83.1 (133)	1.1 (0.7 to 1.6)
	Gestational	14.5 (11)	85.5 (65)	0.9 (0.5 to 1.7)
	Post pregnancy	20.5 (8)	79.5 (31)	1.3 (0.6 to 2.9)
Higher tertile	No diabetes	25.6 (4,093)	74.4 (11,893)	1.00 (Ref)
	Existing	30.5 (94)	69.5 (214)	1.3 (1.0 to 1.6)*
	Gestational	35.2 (82)	64.8 (151)	1.6 (1.2 to 2.1)*
	Post pregnancy	28.8 (53)	71.2 (131)	1.2 (0.9 to 1.6)

**Table 7:** Prevalence of large offspring at age 5 years, stratified by mother’s diabetes status during or after pregnancy (sensitivity analysis).

	No diabetes (95%CI)	Existing diabetes (95%CI)	Gestational diabetes (95%CI)	Diabetes developed post Pregnancy (95%CI)
N	308,070	1,771	4,442	2,694
>90^th^ centile at age 60 months	19.7 (60,683)	24.8 (439)	24.2 (1,073)	23.3 (627)
	(19.6 to 19.8)	(22.8 to 26.9)*	(22.9 to 25.4)*	(21.7 to 24.9)*

Imputation of weight at age 5 years and inclusion of women who were originally excluded as they did not have a full 12 month GP record (in order to accurately classify maternal diabetes category). Combining imputed values with those mother-child pairs originally excluded from our study provided a total of 316,977 mother-child pairs. There was no effect of diabetes on infant weight at age 5 years when adjusting for confounding factors (maternal age, smoking and pre-pregnancy weight) (Table 7 and Table 8). Stratum specific analyses revealed those children born to mothers in the high weight tertile with existing diabetes (OR:1.2 (95%CI 1.0 to 1.6) or gestational diabetes (OR:1.4 (95%CI 1.1 to 1.7) were at a higher risk of being large in comparison to those born to mothers with no diabetes (see Table 9).

**Table 8:** Regression analysis.

Risk factor	Large at age 60 months		
	Odd ratio		95%CI
Pre-pregnancy weight (per kg increase in weight)		1.0	1.0–1.0
Pre-existing diabetes		1.2	0.8–1.4
Gestational diabetes		0.9	0.7–1.2
Diabetes after pregnancy		0.8	0.6– 1.2
Mothers age at delivery		1.0	1.0–1.0
Smoking in pregnancy		0.9	0.9–1.0*

Adjusted odds ratio for large size at age 60 months (sensitivity analysis).

**Table 9:** Odds ratio for having a large infant at age 5 years stratified by prepregnancy weight tertile (comparisons between differing maternal diabetes type and mothers with no diabetes, sensitivity analysis).

Prepregnancy weight		Large child	Non large child	OR (95%CI)
		% (n)	% (n)	
Lower tertile	No diabetes	10.1 (2,257)	89.9 (20,197)	1.00 (Ref)
	Existing	7.8 (8)	92.2 (95)	0.8 (0.4 to 1.6)
	Gestational	9.3 (13)	90.7 (127)	0.9 (0.5 to 1.6)
	Post pregnancy	8 (5)	92 (58)	0.8 (0.3 to 1.90
Middle tertile	No diabetes	16.7 (3,843)	83.3 (19,137)	1.00 (Ref)
	Existing	17.3 (32)	82.7 (153)	1.0 (0.7 to 1.5)
	Gestational	19.6 (37)	80.4 (152)	1.2 (0.8 to 1.7)
	Post pregnancy	19.4 (13)	80.6 (54)	1.2 (0.7 to 2.2)
Higher tertile	No diabetes	26.5 (5,828)	73.5 (16,124)	1.00 (Ref)
	Existing	31 (116)	69 (258)	1.2 (1.0 to 1.6)*
	Gestational	33.2 (151)	66.8 (304)	1.4 (1.1 to 1.7)*
	Post pregnancy	26.6 (62)	73.4 (171)	1.0 (0.7 to 1.3)

## Discussion

### Findings of This Study

We found evidence that diabetes in pregnancy is associated with larger birth weight. However, mothers with existing diabetes were less likely to have a large infant at 12 months. There is limited evidence that diabetes in pregnancy leads to a large weight of the child at age 5 years as only in women in the highest pre-pregnancy weight tertile was there an association between maternal diabetes and a large child. Therefore the influence seen at birth does not last into childhood. The finding that there is an increased risk of macrosomia as a result of foetal exposure to maternal diabetes, is already supported by an extensive field of research establishing maternal glucose as a key determinant of growth and adiposity in the offspring at birth [18]. Findings did not change when adjusting for individual or combined confounding factors whilst controlling for smoking during pregnancy increased the odds of birthing a large baby amongst women with diabetes. Interestingly, in our cohort the infants exposed to normal glucose concentrations in uteris but whose mothers later developed diabetes also had higher birth weights. This finding could be indicative of the effects of a poor maternal lifestyle, which precedes the onset of diabetes, and subsequently exposes the developing foetus to an adverse environment e.g. excess maternal weight and poor nutrition [19].

Type 2- and gestational diabetes have been shown to arise from the same risk factors, concur the same genetic susceptibility and are thus deemed aetiologically indistinct [20]. The estimated risk of women with gestational diabetes later developing type 2 diabetes is between 17–63% (within 5–17 years) [21]. Type 1 diabetes however, is comprised of different genetics, environmental triggers [22] and a more severe glycaemic disturbance [23]. Despite differences in glycaemic disturbance however, a recent systematic review and meta-analysis concluded similar perinatal outcomes e.g. rates of still birth, congenital malformations and neonatal morbidity amongst type 1 and type 2 diabetic pregnancies [23].

We found at age 1 year offspring born to mothers with existing diabetes were less likely to be large in comparison with those born to mothers without diabetes, whilst no association was evident at age 5 years using complete data. This supports Manderson and colleagues [24] who found no differences in offspring BMI at ages 5–11 years despite findings of increasing macrosomia rates and metabolic abnormalities such as higher total cholesterol and fasting insulin levels. Similarly, no differences in adiposity were observed in a cohort of adult offspring [25] or a recent cohort study [26] concluding that maternal type 1 diabetes was not an independent contributor to offspring overweight at ages 2, 5 or 8 years unlike short breastfeeding duration and high birth size. In contrast, findings in multi-ethnic [27] and white [6], [28] populations have reported evidence of foetal programming of later adiposity amongst offspring exposed to existing diabetes in utero. However, the studies that did find an association focused on older children (aged 1.5– 16 [27], 7 [28] and 5–15 [6]) and had small sample sizes (range 35–168 participants).

We did observe an association between gestational diabetes and an increased risk of being large at age 5 years. Our sensitivity analyses using imputed weight also showed an association between existing diabetes and being large at age 5 years. In agreement, findings in multi-ethnic [27] and white [6], [28] populations have reported evidence of foetal programming of later adiposity amongst offspring exposed to diabetes in the uteris. Previous studies [29], [30] reporting a causal relationship between maternal diabetes and offspring obesity have provided a supportive view of the Pedersen hypothesis [31]. Proposing fetal programming as a result of exposure to abnormal glucose levels, such studies suggest that long term anthropomorphic and metabolic alterations occur as a result of maternal diabetes. However, such studies have been previously criticised for their lack of consideration towards other contributing factors including maternal obesity [12].

Examining the effect of maternal pre-pregnancy weight in our study revealed those children born to mothers with gestational diabetes in the middle or highest weight tertile were significantly at risk of being large, a finding in agreement with Boerschmann et al [32]. This was also true of mothers with existing diabetes in our sensitivity analyses. This implies that in terms of diabetes in pregnancy it is an interaction between maternal weight and maternal diabetes that is associated with being large at age 5 years. This concept is further supported by recent studies examining health behaviours of women with gestational diabetes, noting suboptimal physical activity levels [33] and more recently a lower adherence to healthy lifestyle patterns [34].

Our findings suggest that only when maternal diabetes is accompanied by a high pre-pregnancy weight, rather than glucose levels throughout pregnancy alone, the likelihood of a large child is affected. Consequently our study provides limited support for the Pedersen hypothesis [31]. Much of the association is likely to be explained by unmeasured confounding such as ethnicity, socioeconomic status, education and adherence to treatment. We must also acknowledge the role of underlying genetic components. Therefore our findings present very little evidence for an effect of maternal diabetes on childhood obesity warranting further research in order to fully understand the underlying mechanisms.

### Strengths and Limitations

A major strength of the study is the large scale population of which we were able to track both retrospective and prospective health records regarding both mother and child [35]. This enabled us to conduct the study over a 12 year period and include pre-pregnancy weight data. However, inevitable limitations arise when working with routine data. We must acknowledge the potential for misclassification bias with the possibility that our ‘diabetes developed post pregnancy’ group may have been contaminated by women with gestational diabetes mellitus who may have been missed. Furthermore no hyperglycaemia diagnosis codes are available in the routine data. Thus, it is not always clear if glucose values in the database were related to fasting or non fasting test results. Therefore, the identification of this group (hyperglycaemia without diabetes) was deemed unreliable using routine data, leading to the exclusion of this group from our study.

Secondly to increase the sensitivity and capture reliable diagnoses, a large proportion of women who had not been registered with a GP for longer than one year prior to the pregnancy were originally excluded, significantly impacting upon our sample size. Pregnancies excluded due to lack of complete GP records (GP record for less than 12 months before the birth of child) were comparable to those included in the analysis in terms of age (28.5 years vs. 28.3), deprivation (17% vs. 21% in the most deprived ranking) and birth weight (3.34 kg (SD 6.1) vs. 3.4 kg)). However, we did include these women later in the sensitivity analysis.

Thirdly, a large number of children did not have electronic records of weight at age 5. These children were shown to be comparable to those with recorded weights in terms of gender (49% vs. 48.2% female), birth weight (3.37 compared to 3.36 kg) and gestational age (39.30 vs. 39.24). However, those with missing data were more likely to be deprived (p = 0.001) and data is likely to be missing at random depending on data captures of weight at school entry/school rather than any difference in child weight. However, we did use imputed estimates of child weight.

Lastly poor documentation of fields within the electronic records prevented us from assessing the effects of potential confounders e.g. ethnicity, gestational weight gain and infant feeding regimes within our findings. We are therefore unable to rule out the effects of unmeasured confounding. Maternal BMI was only available for half of our study population and infant height measurements are not available meaning we could not look at BMI of child. Therefore our findings should be interpreted with caution.

## Conclusions

Our findings provide insight into the complex relationship between maternal diabetes and the later risk of offspring being large. Indicating an association between maternal diabetes, pre-pregnancy weight and childhood weight, our study demonstrates the need for future research on the multi-factorial parameters surrounding maternal diabetes and offspring size.

## Supporting Information

Table S1 **Explanatory and outcome variables.** A table displaying data source and read codes used for study explanatory and outcome variables.
(DOCX)
