# Supplementary material for: Association of Diabetes in Pregnancy with Child Weight at Birth, Age 12 Months and 5 Years – A Population-Based Electronic Cohort Study
Source: PLoS One. 2013 Nov 13;8(11):e79803. doi: 10.1371/journal.pone.0079803 (PMC3827417; doi:10.1371/journal.pone.0079803)
Supplement: Table S1 — Explanatory and outcome variables. A table displaying data source and read codes used for study explanatory and outcome variables. (DOCX) [file pone.0079803.s001.docx]

Table S1- Explanatory and outcome variables

| **Exposure variable** | **Database** | **Codes used/ Description** |
| --- | --- | --- |
| Diabetes Codes | General Practitioner, National Community Child Health Data, BRECON (type 1 diabetes register) | C10%, Cyu2., Cyu20, L180., L1800, L1801, L1802, L1803, L1804','L1805','L1806', L1807, L1808, L1808, L1809, L180X, L180z, Lyu29 Initial date of diagnosis was also provided. Any diagnosis of type 2 diabetes within 10 months before and up to 2 months after pregnancy was treated as Gestational diabetes. After 2 months of birth delivery, a diagnosis of type 2 diabetes signified mothers who developed diabetes post pregnancy. |
|  |  |  |
| Medication codes | General practitioner | f1%, f2%, fw% |
| **Confounding variables** |  |  |
| Deprivation | National Community Child Health Database | Lower Super Output Area (LSAO) |
|  |  |  |
| Pre-pregnancy weight | General Practitioner | Weight nearest date of delivery recorded within 5 years preceding delivery date (not including pregnancy period). Any BMI values less than 13 kg/m^2^ and greater than 85 kg/m^2^ were disregarded. |
|  |  |  |
| Smoking status | General Practitioner | 1371., 137K, 137N., 137O., 137S., 137T., 1377., 1378., 1379., 137A., 137B., 137F., 137i., 137b., 137C., 137c., 137G. , 137V., 13p.., 13p0., 13p1., 13p2., 13p3., 13p4., 13p5., 745H., 745H4, 8H7i., 8HTK., 9N2k., 137d., 8I39., 1372., 1373., 1374., 1375., 1376., 137a., 137e., 137g., 137H., 137J., 137M., 137P., 137Q., 137R., 137V., 137b., 137C., 137c., 137D., 137d., 137e., 137f., 137G., 137g., 137h., 137Q., 63C5. |
|  |  |  |
| Age | General Practitioner/ National Community Child Health Database | Mother’s date of birth subtracted from date of delivery. Women outside the age range of 12-65 years at the time of delivery were excluded. |
|  |  |  |
| Parity | National Community Child Health Database | Number of children born to mother before birth of child follow-up in the study. |
| **Outcome variables** |  |  |
| Weight at birth | National Community Child Health Database | Values were excluded if weight was outside the range of 1kg to 6 kg’s. A birth weight exceeding 4kg classified a child as large at birth. |
|  |  |  |
| Weight at 12 months | National Community Child Health Database | Closest weight recording between the ages of 6 to 18 months was used. Values were disregarded if below the 0.4^th^ or above the 99.6^th^ centile as recorded in the UK-WHO growth charts. |
|  |  |  |
| Weight at 60 months | National Community Child Health Database | Closest weight recording between the ages of 4 to 6 years was used. Values were disregarded if below the 0.4^th^ or above the 99.6^th^ centile as recorded in the UK-WHO growth charts. |
